# On-Chip High-Finesse Fabry-Perot Microcavities for Optical Sensing and Quantum Information

**DOI:** 10.3390/s17081748

**Published:** 2017-07-31

**Authors:** Mohammad H. Bitarafan, Ray G. DeCorby

**Affiliations:** ECE department, University of Alberta, 9107-116 St. NW, Edmonton, AB T6G 2V4, Canada

**Keywords:** Fabry-Perot, integrated optics devices, microcavities

## Abstract

For applications in sensing and cavity-based quantum computing and metrology, open-access Fabry-Perot cavities—with an air or vacuum gap between a pair of high reflectance mirrors—offer important advantages compared to other types of microcavities. For example, they are inherently tunable using MEMS-based actuation strategies, and they enable atomic emitters or target analytes to be located at high field regions of the optical mode. Integration of curved-mirror Fabry-Perot cavities on chips containing electronic, optoelectronic, and optomechanical elements is a topic of emerging importance. Micro-fabrication techniques can be used to create mirrors with small radius-of-curvature, which is a prerequisite for cavities to support stable, small-volume modes. We review recent progress towards chip-based implementation of such cavities, and highlight their potential to address applications in sensing and cavity quantum electrodynamics.

## 1. Introduction

In its simplest form, the Fabry-Perot cavity (FPC) confines light between two planar and parallel mirrors. Its historical importance to optics and photonics (and in fact physics) is difficult to overstate. Owing to its higher resolving power and light throughput compared to diffraction grating instruments [1], the FPC played a central role in early studies of the fine structure of spectral lines [2]. In the modern era, the FPC (in one form or another) has provided the predominant means for optical feedback and spectral discrimination in lasers. Moreover, the FPC is often employed as an archetype for the description and understanding of other optical resonators (e.g., ring and photonic crystal resonators), which exhibit analogous properties.

An important figure-of-merit (FOM) for the FPC is the finesse (*F*), which essentially quantifies the average number of times that a resonant photon bounces back-and-forth (i.e., the average number of round trips) between the mirrors, prior to being absorbed, scattered, or transmitted out of the cavity. While ultimately limited by the mirror reflectance, the finesse is typically determined in practice by non-idealities such as non-parallelism between the mirrors and the non-plane-wave nature (i.e., divergence) of the incident light [3]. Historically, macroscopic planar-mirror FPCs have been limited to *F* on the order of 100. While quality factor (*Q*) and thus resolving power can be increased by increasing the mirror spacing (i.e., since *Q* ~ *mF*, where *m* is the mode order), this is at the expense of a reduction in free-spectral-range (FSR) and an increase in mode volume (*V_M_*), the latter of which is a critical FOM for many of the applications discussed below [4]. In the past few decades, the development of microelectromechanical systems (MEMS) processes has enabled the scalable fabrication of FPCs on chips. Nevertheless, planar-mirror MEMS-based FPCs are prone to essentially the same finesse-limiting factors as their macroscopic counterparts [5].

Many of the shortcomings of the planar FPC can be mitigated by the use of intentionally curved (often spherical) mirrors, first studied and developed for gas lasers [2]. With appropriate choice of mirror spacing and curvature, the spherical-mirror FPC (one or both mirrors can be curved) can support stable 3-dimensionally confined fields, mathematically described by the Hermite-Gaussian or Laguerre-Gaussian families of modes [6]. As early as 2000, researchers had reported [7] macroscopic spherical-mirror FPCs with *F* > 10^5^, by using ultra-smooth substrates and ultra-low-loss dielectric ‘supermirrors’. However, those cavities were characterized by relatively large mode volumes [4], due to practical tradeoffs between the mirror curvature, aperture, and spacing. Driven by applications in fiber communications, MEMS-based spherical-mirror FPCs were developed in the late 1990s [8], and commercial devices reportedly achieved *F* > 10^3^ [9,10].

As described below, many emerging applications in sensing and quantum information require cavities that simultaneously provide high *Q* and low *V_M_*. Compared to other types of micro-cavities, the ‘air-gap’ FPC provides additional compelling advantages: it is inherently tunable through adjustment of the mirror spacing (i.e., cavity length) and it can provide open-access (for analytes or atomic emitters, etc.) to the high-field regions of the cavity mode, which resides primarily in the space between the mirrors. These factors have driven a significant effort towards the realization of micro-scale, curved-mirror FPCs, using techniques such as focused-ion-beam (FIB) milling [11] or laser ablation [12] to form the curved surfaces. A popular approach, which has the significant advantage of providing a built-in means for light-coupling, is to fabricate one or both mirrors on the end-facet of a single-mode optical fiber [12,13]. Others have employed wafer-bonding approaches combined with precision alignment stages [14]. However, the realization of truly monolithic, curved mirror FPCs with high *F* and low *V_M_* remains at a relatively nascent stage of development [15,16].

In this review, our primary goal is to summarize important applications in sensing and information science that can benefit from open-access FPCs, and in particular to review the role that *Q* and *V_M_* play in determining device performance. We also review recent efforts towards the integration of high *F*, low *V_M_* FPCs on (or at least with) chips containing other functional elements such as electrical and magnetic wiring. Since these efforts are at a relatively early stage of development (and also in the interest of providing appropriate context), both planar-mirror and non-monolithic approaches (e.g., wafer bonding and fiber-on-chip strategies) are included in the discussion.

## 2. Background

**Figure 1:**
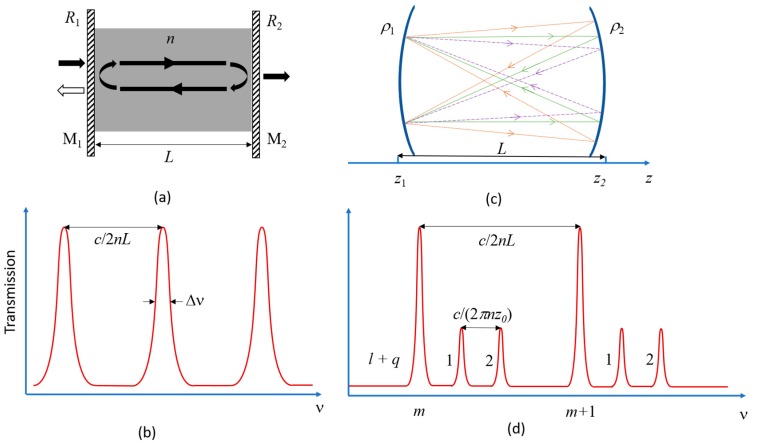
(**a**) Schematic of a Fabry-Perot resonator consisting of parallel mirrors M_1_ and M_2_ with reflectances of *R*_1_ and *R*_2_, respectively, surrounding a cavity medium of refractive index *n* and thickness *L*. Light introduced from one side undergoes multiple reflections, leading to partial transmission from the other side; (**b**) Typical transmission of a planar FPC depicting frequency spacing (i.e., the FSR) of adjacent longitudinal modes and the full-width at half-maximum (Δ*ν*) of an individual modal peak; (**c**) Schematic representation of a curved-mirror FPC with length *L,* where mirrors with radii of curvature *ρ*_1_ and *ρ*_2_ are placed at *z*_1_ and *z*_2_, respectively; (**d**) Typical transmission spectrum of a curved-mirror FPC as a function of mode numbers *l*, *q*, and *m*, where *ρ*_1_, *ρ*_2_* >> L* is assumed.

All optical cavities (optical resonators) ‘trap’ light at one or more resonant frequencies, in the form of standing waves confined by reflective boundaries [4]. The FPC is the archetypal, and arguably also the most important type of, optical resonator. In its most basic form (see Figure 1a), the FPC consists of two plane mirrors M_1_ and M_2_, with reflectance *R*_1_ and *R*_2_, respectively, separated by a medium with refractive index of *n* and length *L*. For an ‘empty’ cavity (i.e., with a non-solid cavity medium), the cavity length can be adjusted (i.e., tuned) by moving one or both mirrors. The resonant condition for the FPC is essentially as follows: the round-trip phase shift for light bouncing back and forth between the mirrors must be a multiple of 2*π*, in order that the infinite set of partial reflections interferes constructively. As described below, FP resonators can be categorized into two groups: planar-mirror and curved-mirror FPCs.

For the planar-mirror FPC (Figure 1a), if we assume that the mirrors have infinite extent in the directions parallel to the medium boundaries, then the problem can be analyzed using plane waves. For the applications discussed here, we shall restrict our attention to normally incident light. Furthermore, the discussion that follows assumes a symmetric cavity (*R*_1_ = *R*_2_ = *R*) and ‘hard-mirror’ boundary conditions. In other words, the phase change on reflection, which results in an effective increase in the cavity length for real mirrors [17], is initially neglected. For example, this is valid for the case of perfect metallic mirrors, where there is a *π* phase-shift at each mirror; thus, the overall phase-shift is 2*π* and can be neglected in that case.

If light in the form of a plane wave is normally incident onto the planar-mirror cavity, it undergoes multiple partial reflections, leading to numerous transmitted and reflected field components. Light travelling a single round trip is subject to a propagation phase shift given by:

(1) $$\delta =\frac{4\pi }{\lambda_{0}}nL,$$

where *λ*_0_ is the wavelength of light in vacuum. The overall (net) transmission can be obtained by a summation over the infinite set of transmitted sub-components, and is expressed [18]:

(2) $$\frac{I_{t}}{I_{i}}=\frac{{\left(1-R\right)}^{2}}{{\left(1-R\right)}^{2}+4R\sin^{2}\left(\delta /2\right)},$$

where *I_i_* and *I_t_* are the total incident and transmitted intensity and *R* is the reflectance of the mirrors. From Equation (2), one can readily find that unity transmission is predicted whenever *δ* = 2*mπ* where *m* is an integer. Incorporating this condition into Equation (1) results in the condition for a resonance frequency:

(3) $$\nu_{m}=m\frac{c}{2nL},$$

where *m* represents the *longitudinal* mode order. Accordingly, the allowed modal resonance wavelengths can be written as:

(4) $$\lambda_{m}=\frac{2nL}{m}.$$

The frequency difference between two consecutive resonance frequencies of mode order *m* and *m* + 1, known as the ‘free spectral range’ (FSR), can be found from Equation (3) [18]:

(5) $$\nu_{f}=\frac{c}{2nL}.$$

This spacing is depicted in Figure 1b.

Neglecting material dispersion and mirror penetration effects, FSR is a constant factor over the entire frequency range; however, in terms of wavelength, FSR is inversely proportional to the mode order:

(6) $$\lambda_{f}=\frac{\lambda_{m}}{\left(m+1\right)}.$$

Two FOMs are used to quantify the frequency selectivity of an optical resonator; finesse (*F*) and quality factor (*Q*). Finesse is defined as:

(7) $$F=\frac{\nu_{f}}{\Delta \nu }.$$

where Δ*ν* is the ‘full width at half maximum’ (FWHM) of the resonance peak. Physically, it represents the average number of round-trips made by a resonant photon before it leaves the cavity. The *Q*-factor is often used for electrical resonance circuits and microwave resonators, and is universally defined as:

(8) $$Q\equiv 2\pi \left(\frac{\mathrm{stored}\;\mathrm{energy}}{\mathrm{energy}\;\mathrm{loss}\;\mathrm{per}\;\mathrm{cycle}}\right).$$

For sufficiently high values, *Q* is conveniently expressed in terms of the resonance frequency and the full-width-at-half-maximum of the resonator line-shape:

(9) $$Q=\frac{\nu_{m}}{\Delta \nu }.$$

Combining Equations (7) and (9), one can readily verify that *Q = mF*. In planar-mirror FPCs, non-parallelism and uncontrolled curvature of the mirrors leads to degradation of *Q* and *F* [3], which can be further reduced by beam walk-off arising from the non-plane-wave nature of a real beam [19]. To avoid excessive walk-off losses, the flat mirrors need to have transverse dimensions that are significantly larger than the input beam. FPCs constructed from curved mirrors can mitigate many of the aforementioned problems. In these cavities, light rays ‘retrace themselves’ as they travel back and forth between the mirrors, which means the mirrors effectively refocus the circulating light (see Figure 1c). 

The modes of a curved-mirror resonator are the beam-like solutions of the paraxial Helmholtz equation, known as Hermite-Gaussian modes. For stable modes, the wavefront curvature at the positions of the mirrors matches the radii of curvature (ROC) of the mirrors (*ρ*_1_ and *ρ*_2_). Applying the phase condition to the Hermite-Gaussian beam yields the resonance frequencies as follows [18,20]:

(10) $$\nu_{l,q,m}=m\nu_{f}+(l+q+1)\frac{\Delta \zeta }{\pi }\nu_{f},$$

where *l*,*q* = 0,1,2,... are *radial* and *azimuthal* mode orders, Δ*ζ* = *ζ* (*z*_2_) − *ζ* (*z*_1_), *ζ* (*z*) = tan(*z*/*z*_0_) is the ‘on-axis longitudinal phase delay’ known as the ‘Gouy phase’ of the beam [18], *z*_0_ is the position of minimum spot size known as ‘beam waist’, and *z*_1_ and *z*_2_ are positions of mirrors M_1_ and M_2_, respectively. Equation (10) indicates that the *longitudinal* modal spacing is independent of the mirror curvatures, and is the same as that for the planar mirror Fabry-Perot. The second term in Equation (10) represents a shift in all resonance frequencies, and is dependent on mirror curvatures. 

It is useful to know the frequency spacing between transverse modes. To this end, we restrict ourselves to short cavities in which *L* << |*ρ*_1_|, |*ρ*_2_|. The frequency spacing for this specific case is given by [6]:

(11) $$\Delta \nu \approx \frac{c}{2\pi nz_{0}}\Delta (l+q).$$

Figure 1d illustrates this spacing for two adjacent transverse modes. 

Mode volume is another factor which is important in applications discussed in the remainder of this paper. The mode volume for a standing-wave associated with the TEM_00_ mode is approximately given by [12]:

(12) $$V_{m}\approx \frac{\pi }{4}w_{0}^{2}L,$$

where *w*_0_ is the waist radius for the mode [12]:

(13) $$w_{0}\approx \sqrt{\frac{\lambda }{\pi }}{\left(L\frac{\rho_{1}\rho_{2}}{\rho_{1}+\rho_{2}}\right)}^{1/4},$$

For the case of plano-concave or half-symmetric cavities where *ρ*_2_ is infinite and thus |*ρ*_1_| << |*ρ*_2_| (as in the case of refs. [15,16,21,22]), the beam waist lies at the planar mirror and is given by the expression:

(14) $$w_{0}\approx \sqrt{\frac{\lambda }{\pi }}{\left(L.\rho_{1}\right)}^{1/4}.$$

Finally, it is worth noting that *Q* and *V_M_* essentially quantify the degree of temporal and spatial confinement, respectively, of the field by the cavity [23].

## 3. FPCs for Sensing Applications

### 3.1. Refractometric Sensors—Background

Optical refractive index (RI) detection is a commonly employed scheme for optofluidic sensing, owing to its potential for label-free, high-precision, and real-time detection. Thanks to their multi-pass nature, optical resonators can be used to enhance light-matter interactions, thereby improving sensitivity. A wide variety of optical resonators have been studied for RI detection including surface plasmon resonators (SPR) [24,25], ring resonators [26,27], fiber-grating-based cavities [28], and photonic crystal cavities [29,30]. In all of these cases, resonant wavelengths are highly sensitive to changes in refractive index within some portion of the mode field. The change in refractive index can be induced by changes in temperature, pressure, etc., or by the presence of some target analyte. For most microcavity sensors, light is confined primarily within a high index medium, for example by total-internal reflection, and only the evanescent portion of the mode field (outside the high index medium) interacts with the target analyte. Resonant cavity sensors of this type, for example whispering-gallery-mode-based sensors [31], can exhibit extremely high *Q* (which correlates with high sensitivity, up to a point, as discussed below). However, these cavities are not suitable for applications where tracking the entire volume of a sample is needed.

There are many sensing problems that can benefit from a stronger interaction between the cavity mode field and the analyte, including sensing of large particles such as cells [32] and sensing within weakly absorbing analyte media. One approach to ‘bulk sensing’ is to use hollow-core waveguides (including hollow fibers), which can simultaneously confine light and a gas- or liquid-phase medium within the same volume [33]. However, these waveguides typically need to be reasonably long (~2 cm) in order to attain a desirable signal-to-noise (SNR) ratio for the detected index change. Alternatively, ‘open-access’ FPC sensors provide a platform for whole-body interactions between samples and a resonant optical field. As in any microcavity, the multi-pass nature of the light propagation can allow for high-SNR detection of minute changes in physical parameters, even for small device dimensions (i.e., mirror spacing of a few microns). Most of the FPC RI sensors reported to date have employed fiber-based cavities, wherein the mirrors were deposited or bonded on the end facets of fibers [34]. However, fiber-based FPCs must be aligned with great precision, and misalignment errors combined with the lack of control over the divergence angle of the output beams can lead to low *Q*-factor [32,35,36,37]. In addition, fiber-based FPCs cannot be easily integrated onto microfluidic chips; several post-fabrication steps are typically required to make them fully compatible for lab-on-a-chip applications. Nevertheless, only a few examples of fully monolithic integrated FPCs for optofluidic RI sensing exist, as briefly reviewed below.

The sensing mechanism in FPC sensors is based on the relationship between the optical path length and the resonant wavelengths of the cavity. Indeed, any change in the optical path length, arising from changes in the refractive index of cavity medium and/or changes in the cavity length (i.e., mirror spacing), will give rise to a shift in cavity transmission peak. Ignoring field penetration into the mirrors, this can be expressed [38]:

(15) $$\frac{\Delta \lambda_{0}}{\lambda_{0}}\approx \frac{\Delta n}{n}+\frac{\Delta L}{L}.$$

Here, *λ*_0_ is a resonant (i.e., peak transmission) wavelength of the cavity. While exceptions exist, most FPC sensors employ an approximately fixed mirror spacing, so that changes in peak wavelength can be directly correlated to changes in the refractive index of the cavity medium. This is the scenario commonly known as refractometric (refractive-index, RI) sensing. A common FOM for refractometric sensors is the detection limit (DL), defined as the minimum detectable refractive index change (i.e., DL = Δ*n*_min_). It can be expressed as DL = *R*/*S* [31] where *R* and *S* are the resolution and sensitivity, respectively. The sensitivity is defined as *S* = Δ*λ*_0_/Δ*n,* and is simply a measure of the resonant wavelength shift per unit refractive index change. To first-order, we can write *S* = Γ*λ*_0_*/n_eff_* [31], where Γ is the optical confinement factor within the analyte volume (i.e., the fraction of the optical mode interacting with the target analyte) and *n_eff_* is an effective index for the resonant optical mode. For most sensors that employ evanescent-mode-field interactions (with SPR sensors being an exception [31]), sensitivity is relatively low because Γ is low. For the FPC sensor, Γ can approach 1 as implied by Equation (15) (which neglects field penetration inside the mirrors), so that *S* can be significantly improved. Resolution is defined as the minimum spectral shift that can be accurately detected (i.e., *R* = Δ*λ_min_*), and is ultimately limited by noise in the detecting apparatus.

Sensitivity (as defined above) has been commonly employed as a comparison metric for refractometric sensors. However, as discussed by Hu et al. [39], this quantity fails to account for the role of microcavity parameters such as *Q* and *F* in determining the limit of detection. Aside from cavity parameters, DL also depends on the particular characteristics (e.g., wavelength resolution, noise characteristics, and noise bandwidth) of the detection instrumentation used for readout. In general, both intensity noise (e.g., due to photodetector noise) and wavelength noise (e.g., due to wavelength repeatability of the interrogation instruments) will impact the overall system performance. Hu suggested an alternative FOM, called the time-normalized sensitivity (analogous to the parameter ‘detectivity’, used to characterize photodetectors) [39]:

(16) $$S^{*}=\sqrt{\frac{\Delta f}{N}}\frac{1}{\Delta n^{*}},$$

where Δn^*^ is the refractive index change that produces a signal-to-noise ratio of unity, and Δ*f*/*N* is called the equivalent noise bandwidth of the detection system. For example, in a wavelength interrogation scheme using a tunable laser source or scanning spectrometer (see below), Δ*f* is the single-measurement noise bandwidth and *N* is the number of discrete wavelength values at which measurements are made. Thus, S^*^ is a FOM that encapsulates characteristics of both the device (i.e., the optical resonator) and the peripheral optical and electronic components (i.e., laser, detector, etc.).

Readout of microcavity sensors is typically achieved using *intensity interrogation*, where the change in transmission of a fixed-wavelength laser source is monitored, or *wavelength interrogation*, where the peak wavelength is continuously monitored by scanning a tunable laser or spectrally selective detector across the microcavity resonance. For a given scheme, it is possible to define a device-specific figure-of-merit (*FOM_D_*), which is determined solely by the characteristics of the microcavity (and not by the particular instrumentation used for interrogation). As an example, in a wavelength interrogation scheme (which is most common) operating in an intensity-noise-limited regime [39]:

(17) $$S^{*}\propto FOM_{D}=\frac{Q\Gamma \lambda_{0}}{n_{g}},$$

where *Q* is the cavity quality factor, *λ*_0_ is the peak (resonant) free-space wavelength, and *n*_g_ is the group refractive index of the cavity mode. Thus, the practical limit of detection for a microcavity sensor scales inversely with both *Q* and Γ, at least up to a point. For *Q* exceeding some maximum value (typically 10^5^ − 10^6^ [39,40]), wavelength noise (in part arising from thermally induced variations in the resonant wavelength of the microcavity [31,41]) becomes the limiting parameter. In other words, practical limitations of interrogation instruments (e.g., the wavelength repeatability of tunable lasers, temperature variations in the microcavity, etc.) set an upper limit on the desirable microcavity *Q*, with further increases providing no significant improvement in the detection limit [31,39,40,42]. Thus, while other microcavities can achieve higher *Q* values, the spherical-mirror FPC can easily provide the values required to maximize S*, in addition to providing near-unity Γ. It should be also noted that, while Equation (17) does not capture the impact of mode volume directly, a reduction in *V_M_* is typically favorable for sensing applications, since it implies a smaller probed analyte volume (i.e., detection of a smaller number of molecules, as discussed further below). Given its potential for moderate-to-high *Q*, high Γ, and low *V_M_*, the spherical-mirror FPC is an intriguing candidate for applications in sensing. Efforts towards this goal have recently been described in the literature, and are briefly summarized in the following paragraphs.

### 3.2. Chip-Based FPCs for Refractometric Sensing

Early work on volume refractometric FPC sensors employed relatively low finesse, planar-mirror FPCs, typically embedded within or on the end-facets of optical fibers. For example, Song et al. [32] used gold-coated fiber end-facet mirrors, hybrid-integrated onto a PDMS microfluidics platform, for the detection of single living cells. A similar refractometer was demonstrated by Domachuk et al. [28], but with fiber Bragg grating mirrors augmented by graded-index fiber sections to provide light collimation between the fiber end facets. Chin et al. [43] also reported a similar device, and demonstrated optical trapping of a live cell within the cavity region. The same group later added liquid microlenses [44] for collimation of the light within the cavity. A related approach, but with integrated, vertical Bragg mirrors fabricated by deep reactive ion etching (DRIE) of silicon was described by St-Gelais et al. [45]. They reported a sensitivity of 907 nm/RIU (RI units) and DL ~1.7 × 10^−5^ RIU. The *Q*-factor of their FPC (~400) was limited by surface roughness and non-parallelism of the mirror sidewalls.

Other researchers have embedded vertical planar-mirror FPCs directly into microfluidic chips by using wafer-bonding processes. For example, Shao et al. [46] formed a dielectric-mirror FPC in a glass-based microfluidics platform, and applied it to the refractometric sensing of lymphocytes. A similar wafer-bonding strategy has been explored by a research group in China [47,48,49]. In their early work, wet etching on silicon-on-insulator (SOI) wafers was used to fabricate on-chip optofluidic channels, where the oxide layer functions as the etch-stop layer leading to smooth microchannel surfaces for mirror deposition. FPCs with mirror reflectance ~75% and *Q* ~ 861 were reported, enabling sensitivity of 1100 nm/RIU and DL ~1.1 × 10^−5^ [47]. Aiming to improve SNR and resolution, an optical differential detection (ODD) method was later explored by the same group. The method is based on the spectral measurement of two cavities, one of which is filled with DI water as the reference. The refractive index change is monitored by tracking the power level at a wavelength tuned to the steepest point of the transmission spectrum associated with the water filled cavity. DL of 5.5 × 10^−8^ was experimentally demonstrated [48]. In later work, surface roughness was further reduced by utilizing glass substrates, with the additional benefit that the operating wavelength of the refractometer could be extended to visible and ultraviolet regions. DL down to 2.0 × 10^−9^ RIU was reported for the all-glass biosensor fabricated by face-to-face bonding of HF-etched Ta_2_O_5_/SiO_2_ Bragg coated glass substrates, with noise reduced by concurrent use of ODD and phase lock-in detection (PLD) [49].

**Figure 2:**
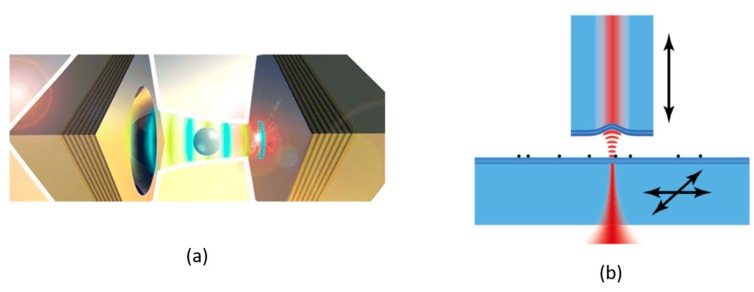
(**a**) Schematic representation of an open-access microcavity with a flat and a concave mirror. A nanoparticle in solution, trapped inside the optical mode is shown [51]; (**b**) Conceptual diagram of a laser-machined fiber with multilayer mirror on its end along with a flat mirror serving as a sample holder; black dots represent nanoparticles located on the flat mirror [52].

Only recently have researchers begun to explore the use of curved-mirror FPCs for refractometric sensing [50]. In addition to enabling much higher *Q* than their planar-mirror counterparts, a primary motivation for this approach is that it can significantly reduce the volume of the analyte needed to achieve a given sensitivity, since it employs low-*V_M_* modes. For example, Trichet et al. [38] employed simultaneous tracking of resonance wavelengths in arrays of wafer-bonded, half-symmetric cavities, and achieved DL ~3.5 × 10^−4^ RIU correlated with the presence of ~10^5^ glucose molecules in a 54 fL detection volume. The same group subsequently reported [51] the trapping and sensing of single nanoparticles, using spherical-mirror FPCs with *Q* ~18,000 and *V_M_* ~1.7 *λ*^3^ at 560 nm wavelength (see Figure 2a). In related work, Mader et al. [52] formed a high-finesse cavity using a spherical mirror on a fiber end-facet, which could be scanned to image and characterize nanoparticles attached to the opposite (planar) mirror of the cavity (see Figure 2b). Finally, Kelkar et al. [53] used a low-*V_M_* spherical mirror cavity to sense single nanoparticles. While at an early stage of development, spherical-mirror FPC sensors hold great promise for the detection of small particles, possibly even down to the single molecule level [38,50]. The key FOMs of several reported FPCs used for refractometric sensing are summarized in Table 1.

### 3.3. Open-Access FPC Lasers

Monolithic integration of laser cavities within lab-on-a-chip (LOC) systems is a long-standing goal in the microfluidics research community. For example, monitoring of laser emission from such a cavity is a promising sensing modality in its own right [54]. The vast majority of lasers employ the FPC as an optical feedback element. The FPC, or any optical cavity, plays a dual role in the laser. First, it enables the build-up of a high photon density within the optical gain medium, so that the stimulated emission rate can greatly exceed the spontaneous emission rate. Second, it acts as a frequency-selective element, determining one or more frequencies (within the gain bandwidth of the laser medium) at which lasing will occur. An early report of a FPC-based microfluidic laser was the work by Helbo et al. [55], which employed a relatively low-finesse cavity formed by wafer bonding of planar gold mirrors surrounding a microfluidic channel. Pulsed lasing was achieved, but with relatively high pump power requirements.

Generally speaking, there is an ongoing drive towards the development of small-scale lasers, typically enabled by photonic crystal or plasmonic resonator structures [56]. The goal is to produce lasers that can be embedded within LOC systems or even within biological systems. While such lasers typically generate relatively low output power, of greater importance is the fact that they might operate with relatively high efficiency and consume relatively low pump power [56]. A key FOM for the miniaturization of lasers is the minimum required threshold gain, which can be approximated [56,57]:

(18) $$G_{TH}\approx \frac{2\pi n}{Q\Gamma \lambda },$$

where Γ is the energy confinement factor (i.e., the overlap factor) of the laser mode with the active gain medium and *n* is the refractive index of the cavity medium. As per the discussion above, the spherical mirror FPC can simultaneously provide moderate-to-high *Q* and Γ ~ 1. Moreover, the possibility for low *V_M_* implies low pump energy requirements [56] and other benefits mentioned below.

**Figure 3:**
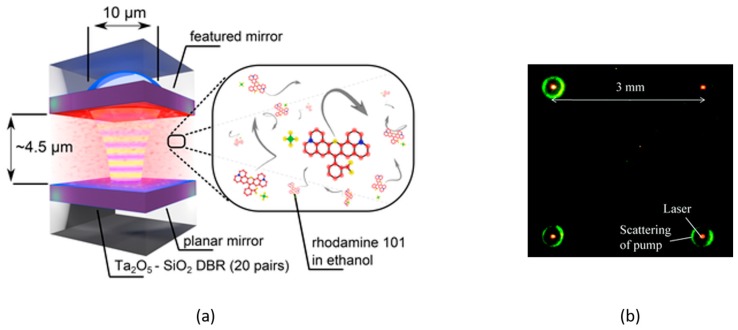
(**a**) Schematic representation of an open-access microcavity with a flat and a concave mirror filled with a rhodamine 101 solution [59]; (**b**) Simultaneous laser emission from four spherical mirror FPCs [22].

The spherical-mirror, open-access FPC is emerging as a promising new platform for small-scale lasers, and is inherently well-suited for the implementation of microfluidic laser systems. An early example was the work by Patel et al. [58], who used a colloidal CdSe-based gain medium in a hybrid-assembled FPC chip. The same research group subsequently reported continuous-wave (CW) operation for similar laser cavities, using organic dye gain media. Typically, microfluidic pumping is required to achieve CW operation from dye-based lasers, since dye media are subject to rapid photobleaching effects. Conversely, Coles et al. [59] were able to achieve CW operation without pumping, owing to the diffusion of organic molecules into and out of the small *V_M_* gain region of their spherical-mirror FPC (see Figure 3a). In parallel with these efforts, a group in China [22,60] has recently reported low-threshold dye lasers based on high-finesse (*F* ~ 10^3^) spherical-mirror FPCs fabricated using a wafer-bonding approach (see Figure 3b). The field of spherical-mirror, open-access microcavity lasers is in its infancy, and promises to yield many exciting results in the coming years.

## 4. FPCs for Cavity Quantum Electrodynamics Applications

### 4.1. CQED—Overview

Technologies that directly exploit quantum wave-functions are widely expected to lead to significant advances in computing [61], secure communications [62], sensing [63], and metrology [64]. Nevertheless, the current state of the art is best summed up as a “grand scientific challenge” [65]. Quantum technologies are predicated on:

(i) the ability to isolate quantum particles (e.g., atoms) from the external environment—i.e., to preserve quantum coherence on time-scales that are technologically useful, and;
(ii) the ability to address, control, and store quantum states, and to build scalable systems with large numbers of quantum particles (‘qubits’).

Optical cavities are firmly established as one of the most important tools for quantum information technologies [4,66]. An optical cavity can greatly enhance the inherently weak interactions between light and matter, and effectively isolate an atom-photon system from the external environment [66,67]. The study of atom-photon interactions in cavities is the domain of cavity quantum electrodynamics (CQED). CQED is expected to be a key enabler of a future ‘quantum internet’ [68], predicated on devices that mediate quantum entanglement between photons and atomic emitters [68,69]. In such a network, quantum states (i.e., qubits) are stored and processed at quantum nodes and optical connections enable nodes to distribute quantum entanglement across the network. It has been suggested that these networks might utilize arrays of tunable, open-access optical cavities on a single chip, into which atoms can be injected and trapped (for example, using magnetic or optical trapping methods) [66,69].

**Figure 4:**
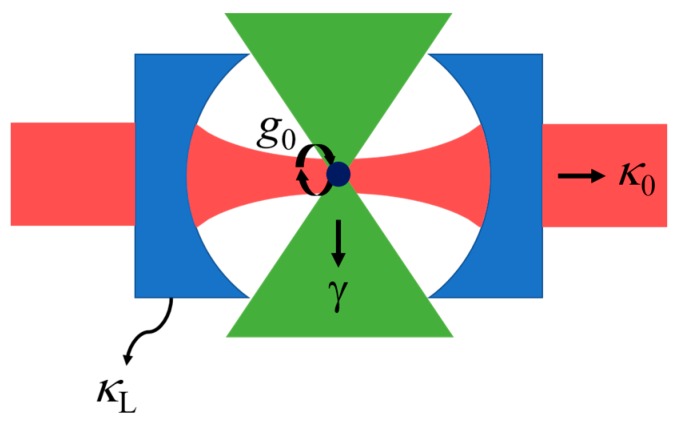
Schematic representation of a CQED system encompassing a single atom inside an FPC.

A canonical Fabry-Perot-based CQED system is depicted in Figure 4. Aside from being the archetypal CQED system, the curved-mirror FPC offers the same unique advantages mentioned in Section 1 (i.e., tuning and open access to the cavity field for placement of atomic emitters). It is worth noting that photonic crystal cavities with air defect modes can also provide open access to the cavity mode field [70,71,72]. Excellent and comprehensive reviews of CQED are available [12,66]. After a brief summary of a few key principles, our main goal below is to provide a survey of recent experimental work aimed at the construction of chip-integrated FPC arrays for CQED. 

Interactions between atoms and photons within a cavity are governed by three rate parameters (see Figure 4): the non-resonant decay rate of the atomic dipole (*γ*) (i.e., the rate of atomic decay by emission into all modes other than the resonant cavity mode of interest), the photon decay rate of the cavity (*κ*), and the atom-cavity coupling rate (*g*_0_). In keeping with most of the CQED literature [66,68], we define *κ* and *γ* as the half-width-at-half-maximum (HWHM) of the corresponding resonance line-shape plotted versus angular frequency. Thus, from the discussion in Section 2, it follows that the cavity decay rate can be expressed [66]:

(19) $$\kappa =\frac{\Delta \omega }{2}=\frac{1}{2\tau_{P}}=\frac{\pi c}{2LF},$$

where *τ_P_* is the photon lifetime of the cavity mode. As depicted in Figure 4, the decay rate can be divided into two parts, *κ* = *κ*_0_ + *κ*_L_, where *κ*_0_ is the rate of photon emission into a desired output channel (typically by transmission through one mirror) and *κ*_L_ is the rate of photon loss (by transmission through the opposite mirror and by scattering and absorption in both mirrors).

The non-resonant decay rate *γ* encapsulates all processes, other than emission of a photon into the resonant cavity mode, by which the excited atom can relax towards its ground state. In the two-level-atom approximation, and neglecting the possibility of non-radiative relaxation (e.g., due to collisions), *γ* is determined by the rate of spontaneous emission into non-resonant modes [66]:

(20) $$\gamma =\left(1-\Delta \Omega /4\pi \right)\cdot \left(\Gamma_{21}/2\right).$$

Here, ΔΩ is the effective solid angle spanned by the resonant cavity mode, Γ_21_ = 1/*τ*_21_ is the free-space spontaneous emission rate (i.e., the Einstein A_21_ coefficient), and *τ*_21_ is the free-space spontaneous emission lifetime for the atomic transition. For conventional macroscopic FPCs, ΔΩ is typically small, so that *γ* is nearly identical to half of the free-space decay rate of the atom. Wavelength-scale FPCs, however, can inhibit emission into non-resonant modes, as discussed further below.

The third parameter, the atom-cavity coupling rate, is essentially governed by the electric-dipole interaction between the atom and the cavity vacuum field (i.e., the zero-point fluctuation of the electromagnetic field [23]). For a single two-level atom placed at the location of the maximum electric field of the cavity mode in an otherwise empty cavity, and with the cavity resonance tuned to match the atomic transition, *g*_0_ can be expressed [23,66]:

(21) $$g_{0}=\sqrt{\frac{\mu^{2}\omega }{2\varepsilon_{0}\hbar V_{M}}},$$

where *μ* is the electric dipole moment for the transition (assumed here to be aligned with the electric field polarization vector of the cavity mode [68] ) and *V_M_* is the volume of the fundamental mode (see Section 2). Many fundamental studies of quantum coherence and entanglement are predicated on achieving so-called ‘strong-coupling’ conditions, in which the atom-cavity coupling rate exceeds both the cavity and atomic decay rates, i.e., *g*_0_ >> (*κ*,*γ*), where (*κ*,*γ*) represents the larger of *κ* and *γ*. In this regime, the atom and the cavity can exchange energy through repeated photon emission and absorption in a reversible fashion. For a single atom and a cavity initially in its vacuum state (no photons in the resonant mode), this energy exchange oscillates at the single-photon Rabi frequency Ω_R_ = 2·*g*_0_ [66]. In the frequency domain, this exchange manifests as a splitting of the cavity resonance, which becomes a double-peaked cavity transmission (vacuum-Rabi splitting [23]) with peaks centered at *ω* ± *g*_0_. Aside from fundamental physics studies, strong-coupling CQED might enable a range of basic building blocks that rely on the observation of resolved coupled-system resonances (such as devices for coherent control and read-out of quantum states [66]). Equation (21) indicates that for a given atomic transition, reduction of mode volume is the critical requirement for increasing *g*_0_ and thus for achieving strong-coupling conditions. To date, the practical implementation of strong-coupled atom-cavity systems, especially in a scalable format, remains a significant challenge.

Fortunately, many important applications of CQED reside in the ‘weak-coupling’ regime, with *g*_0_ << (*κ*,*γ*). In this regime, an important dimensionless factor, known as the single-atom cooperativity, is defined as follows [66]:

(22) $$C=\frac{g_{0}^{2}}{2\kappa \gamma }.$$

The inverse of the cooperativity, *N*_0_ = 1/*C*, is termed the critical atom number [4] and can be interpreted as the number of atoms required to significantly modify the cavity field [68]. For the case that *C* << 1, the interaction between the atom and the electromagnetic field is not significantly modified by the cavity. In contrast, weak-coupling conditions that satisfy *κ* >> *g*_0_ >> *γ* and *C* >> 1 are of great interest, and characterize the so-called “Purcell” regime. In this regime, photon emission is irreversible but nevertheless greatly altered relative to the free-space situation, due to modification of the photonic density of modes available to the emitting atom. The Purcell effect can be used to tailor and control spontaneous emission from an atom, such as by causing preferential emission into a desired resonant mode of the cavity [4]. It is the key parameter of interest for a range of novel light sources, including threshold-less lasers and single-photon sources.

In the Purcell regime, the cavity alters the density of photonic modes in a frequency-dependent fashion. Thus, depending on whether the atomic tranisition is resonant with a cavity mode or not, the rate of radiatve emission is enhanced or suppressed, respectively, relative to the free-space rate. This modification was historically treated by Purcell using a semi-classical approach, with the essential results subsequently confirmed by rigorous quantum mechanical treatments [23]. For example, assuming an atom located at a field maximum (in an otherwise empty cavity, such that refractive index *n* =1) and with its electric dipole aligned to the cavity mode field, and also assuming the atomic transition is exactly matched to a cavity resonance and with *γ* < *κ* [53,73], then the enhancement of the radiative decay rate is given by the so-called Purcell factor:

(23) $$F_{P}\equiv \frac{\gamma_{C}}{\left(\Gamma /2\right)}=\frac{3}{4\pi^{2}}\frac{Q}{\left(V_{M}/\lambda^{3}\right)},$$

where *γ_C_* is the rate of emission into the cavity mode at resonance. A high Purcell factor is of great interest for the realization of efficient quantum light sources (e.g., low threshold lasers and single-photon sources), and requires a combination of high *Q* and low *V_M_*. In light of this, it is not surprising that the Purcell factor can be directly related to the single-atom cooperativity from above. For example, using the free-space value for *γ* (i.e., *γ = Γ/2 = μ*^2^*ω*^3^*/6πε*_0_*ħc*^3^ [23]), and assuming a perfectly resonant and aligned atom as above, it follows that *F_P_* = 2·*C* [66]. A related FOM is the spontaneous emission coupling factor [66]:

(24) $$\beta =\left(\frac{\kappa_{0}}{\kappa }\right)\cdot \frac{F_{P}}{F_{P}+1},$$

which quantifies the fraction of emitted photons that are coupled into the desired output mode. *β* is a key efficiency metric for low-threshold lasers (see Section 3) and especially for single-photon-sources [66], and can approach unity for low-loss, high-*C* cavities. For a range of CQED applications, Equations (23) and (24) show that key requirements for the cavity are a small *V_M_* (thus large *g*_0_) and a small excess photon loss (i.e., *κ*_0_ >> *κ*_L_). Furthermore, for full control of atomic emission, *κ* >> *γ* is required. In the case of solid-state emitters, which have relatively large *γ*, this implies that small *V_M_* is particularly critical (i.e., since increased *κ* implies reduced *Q*) [53,73].

### 4.2. Chip-Based FPCs for CQED

Early work on CQED employed macroscopic cavities, constructured from ultra-low loss dielectric mirrors deposited on super-polished spherical substrates. Finesse as high as ~10^6^ was achieved [7], but the relatively large mirror ROC and aperture typically implies higher-than-desirable mode waist and volume for these cavitites. Moreover, this approach is not particularly scalable, due to the high cost and complexity of cavity fabrication and alignment. These limitations subsequently spurred efforts towards the miniaturization of FPC cavities, typically through the use of micro-machining techniques to form atomically smooth, small-ROC curved surfaces. In many cases, the FPC is constructed with one or both mirrors bonded [74] or deposited [12] directly on the end facet of a single-mode optical fiber, which facilitates direct optical coupling between the cavity mode and the fiber mode. Nearly all cavities reported to date were constructed by aligning two separately deposited mirrors (i.e., on two separate fibers or substrates), and thus employ non-monolithic processes. This can hinder scalability, and also creates challenges with respect to cavity noise and stability [14]. In the following, we briefly summarize micro-machining approaches that have been used to fabricate hybrid-integrated cavities, with some emphasis on recent efforts aimed at the eventual monolithic integration of mirrors and cavities with other functional devices.

**Figure 5:**
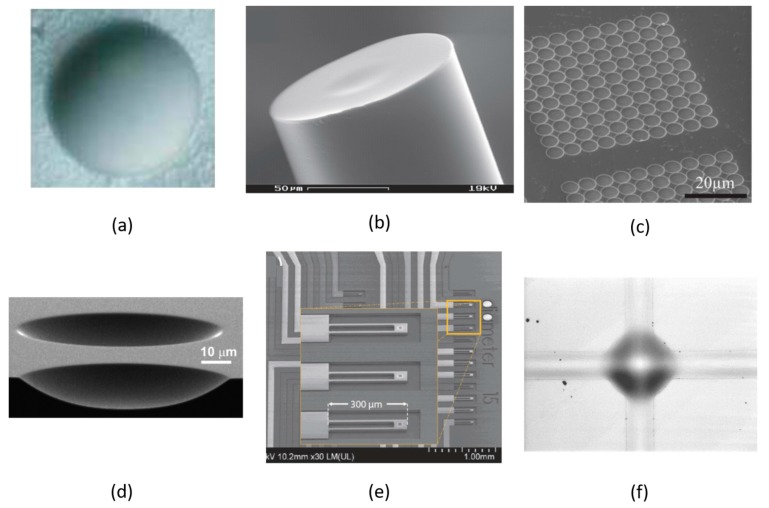
Microscope images of (**a**) a dimple with diameter of 200 μm formed by a bubble-trapping method [76]; (**b**) a laser machined fiber end facet [12]; (**c**) array of concave features made by FIB milling [11]; (**d**) two silicon micro-mirrors fabricated by dry etching [78]; (**e**) array of cantilever based micro-mirrors [79], (**f**) and a microscope image of a waveguide connected buckled-dome microcavity. The dome diameter is 100 μm [80].

A wide variety of micro-machining approaches have been employed to form small ROC mirrors. For example, early work by Prakash et al. [75] used electrochemical growth on a template of latex microspheres to form gold-based hemispherical mirrors. Cui et al. [76] employed a bubble-trapping method to form hemispherical surfaces on a glass substrate, onto which a dielectric mirror was deposited (Figure 5a). Steinmetz et al. [74] detached dielectric mirrors previously deposited on a spherical surface (ball lens or microlens) and glued them to the end of an optical fiber. Recently, however, the most popular techniques have involved standard micro-machining techniques such as CO_2_ laser ablation (Figure 5b) [12,77], focused ion beam (FIB) milling (Figure 5c) [11], and dry etching (Figure 5d) [78].

The etching approach was pioneered by Trupke et al. [81], and has the advantage of enabling the parallel fabrication of multiple concave surfaces. A two-cavity ‘array’ of this type was used for atom detection and photon generation [82]. Biedermann et al. [78] etched ultrasmooth surfaces in silicon to construct cavities with finesse as high as 64,000. FIB-based mirrors were originally reported by Dolan et al. [11]. While inherently a serial process, FIB milling can be used to fabricate large arrays of concave surfaces on a chip, with ROC as small as 5 μm or less and tailored morphology [38]. FIB-fabricated mirrors have enabled cavity finesse as high as 40,000 [83]. Laser-ablation techniques are generally able to produce the smoothest concave features, owing to the melting and reflow of the machined glass surface. Muller et al. [13] achieved a finesse >10^5^, and similar results have been reported by others [12,83]. Laser-ablated surfaces tend to be somewhat non-spherical, however, which can lead to mode-coupling loss [84] and polarization-dependence [85]. Moreover, control over the morphology, especially the relationship between feature depth and ROC, requires additional processing steps [86].

As mentioned, nearly all FPC micro-cavities developed for CQED to date have involved hybrid integration strategies. Nevertheless, some notable efforts towards monolithic integration (of at least one mirror) have been made. Purdy et al. [87] integrated a high-reflectance mirror with on-chip wiring for magnetic trapping of cold atoms and for temperature stabilization. They completed the cavity by hybrid alignment of a macroscopic curved mirror, and achieved a finesse ~ 2 × 10^5^. In another widely cited work [77], Colombe et al. integrated a fiber-based spherical microcavity onto a similar ‘atom’ chip containing magnetic trapping circuitry. Another notable work is that by Derntl et al. [79], in which curved mirrors (fabricated via dry etching) were integrated onto an array of individually tunable cantilevers on a silicon MEMS chip (see Figure 5e). Cavities were formed by mating the cantilever array with flat mirrors formed on the end facets of an array of single mode fibers.

These examples illustrate that many options exist for hybrid integration of low *V_M_*, high *Q* open-access cavities with other chip-based componentry. However, there are few options that promise fully monolithic integration of tunable high-finesse, open access microcavities on chips. Our group has reported a thin-film buckling approach [15,16,21,88] with potential to address this latter point. We’ve shown that circular delamination buckles can be controllably formed within a multilayer thin film stack, and that these features can behave as half-symmetric Fabry-Perot cavities. The buckling self-assembly produces cavities with a high degree of geometrical perfection, characterized by cylindrically symmetric Laguerre-Gaussian modes and reflectance-limited finesse. Recently, Potts et al. reported arrays of these microcavities [16] with SiO_2_/Ta_2_O_5_ Bragg mirrors, and exhibiting *F* ~ 3500 and mode volume ~35*λ*^3^. These cavities were designed for operation near 780 nm wavelength, and could be thermally tuned to the D2 transition of Rb. While the buckled cavities are inherently closed features, open access was demonstrated by FIB milling holes into the upper mirror. Even more recently, we reported Si/SiO_2_-based cavities operating in fundamental mode regime with *V_M_* ~ 1.3*λ*^3^ at 1550 nm wavelength and *Q* ~ 1800 [80]. These parameters correspond to a Purcell factor *F_P_* ~100, making these cavities of interest for CQED applications. Finally, that work also reported fabrication of channel-connected cavities by the same monolithic process (see Figure 5f). Those cavities retain good optical properties while gaining an ‘open-access’ characteristic.

In summary, tremendous progress has been made towards the integration of high *Q*, low *V_M_* FPCs at the chip scale. However, most of the reported work involves hybrid assembly of the FPCs, using precision alignment stages. The monolithic integration of complete FPC-based CQED systems (i.e., full cavities along with magnetic control functions, etc.) onto single chips remains a challenge, and an intriguing avenue for future research. The key FOMs of several reported on-chip FPCs for CQED applications are summarized in Table 2.

## 5. Conclusions

We have reviewed the current state-of-the-art for chip-based, curved-mirror, Fabry-Perot cavities with applications in refractometric sensing, microlasers, and cavity QED. We have furthermore discussed the role of cavity finesse (or *Q*-factor) and mode volume in the performance of FPCs for the aforementioned applications, emphasizing the importance of high finesse and small mode volume. A high *Q*, small mode-volume FPC is predicated on 3D confinement of light using intentionally curved mirrors, which can mitigate finesse-limiting defects associated with conventional planar-mirror FPCs.

Various micro-machining techniques, such as FIB milling, CO_2_ laser ablation, or isotropic etching, have been utilized to fabricate high-curvature surfaces at the micro-scale, either on the end facet of an optical fiber or on a wafer. These methods have recently been employed in the construction of curved-mirror FPCs for each of the application areas mentioned above; an overview of key experimental work was provided. In the vast majority of cases, the FPCs were implemented by means of hybrid assembly and positioning techniques, which creates challenges with respect to cost, stability, and scalability. There is a need for monolithically integrated, curved-mirror, open-access FPCs on chips, which will likely require that concepts from the micro-optical-electromechanical systems (MOEMS) literature [8] be adapted for requirements in sensing and CQED. This is a relatively unexplored approach and should be an interesting avenue for future exploration.

## Figures and Tables

**Table 1:** Summary of chip-based FPCs used for refractometric sensing.

Ref.	Mirror Type	Mirror Material	Wavelength (nm)	*Q*	*F*	*S* (nm/RIU)	DL (RIU)	*V_M_* (λ^3^)
[32]	Planar	Au	1550	330	5.2	-	1.4 × 10^−3^ *	-
[28]	Planar	FBG	1550	-	-	6.825	2.7 × 10^−3^ *	-
[43]	Planar	FBG	1258	-	-	32 *	0.001	-
[44]	Planar	Au	1275	600 *	18.79	960	0.01	-
[45]	Planar	Si/Air	1550	400	9 *	907	1.7 × 10^−5^	-
[46]	Planar	Au	890	900 *	30	-	-	-
[47]	Planar	Ta_2_O_5_/SiO_2_	1550	861	-	1100	1.1 × 10^−5^	-
[48]	Planar	Ta_2_O_5_/SiO_2_	1550	600 *	14 *	-	5.5 × 10^−8^	-
[49]	Planar	Ta_2_O_5_/SiO_2_	1550	875	3	650 *	2.0 × 10^−9^	-
[38]	Curved	TiO_2_/SiO_2_	640	-	1000	-	3.5 × 10^−4^	-
[50]	Curved	TiO_2_/SiO_2_	640	105	4000	-	3.5 × 10^−4^	3.8
[51]	Curved	TiO_2_/SiO_2_	640	18000	4500 *	-	-	1.7
[52]	Curved	Ta_2_O_5_/SiO_2_	780	1.57 × 10^6^	57,000	-	-	100 *
[53]	Curved	TiO_2_/SiO_2_	745/785	300	70	-	-	0.8

* Estimated from related data in the paper.

**Table 2:** Summary of chip-based, curve-mirror FPCs proposed for CQED.

Ref.	Fabrication Method	Mirror Material	Wavelength (nm)	*Q*	*F*	*V_M_* (*λ*^3^)	*C*
[74]	Deposition on microlens	TiO_2_/SiO_2_	780	-	1000	1260	2.1
[75]	Self-assembled templating	Au	747 *	300	15	2.1	-
[76]	Bubble trapping	TiO_2_/SiO_2_	750	-	200	74 *	-
[77]	Laser ablation	Ta_2_O_5_/SiO_2_	780	-	37,000	-	145
[78]	Dry etching	Ta_2_O_5_/SiO_2_	780	-	64,000	-	200
[81]	Wet etching	Au	780	10^6^	6000	-	39
[11]	FIB milling	ZrO_2_/SiO_2_	637	10^4^	460	8.5	10 *
[13]	Laser ablation	Ta_2_O_5_/SiO_2_	920	3.3 × 10^6^	1.5 × 10^5^	51	-
[86]	Laser ablation	Ta_2_O_5_/SiO_2_	637	10^5^	2.5 × 10^4^	-	-
[87]	Dry etching	Ta_2_O_5_/SiO_2_	780	1.4 × 10^7^ *	2 × 10^5^	-	50
[16]	Buckling delamination	Ta_2_O_5_/SiO_2_	780	-	3500	35	32
[80]	Buckling delamination	Si/SiO_2_	1550	1800	1800	1.3	50

* Estimated from related data in the paper.
